# Investigation of Reaction Forces in the Thoracolumbar Fascia during Different Activities: A Mechanistic Numerical Study

**DOI:** 10.3390/life11080779

**Published:** 2021-08-01

**Authors:** Khaled El-Monajjed, Mark Driscoll

**Affiliations:** 1 Musculoskeletal Biomechanics Research Laboratory, Department of Mechanical Engineering, McGill University, Montréal, QC H3A 0G4, Canada; khaled.el-monajjed@mail.mcgill.ca; 2 Orthopaedic Research Laboratory, Research Institute MUHC, Montreal General Hospital, Montreal, QC H3G 1A4, Canada

**Keywords:** finite element analysis, low back pain, biomechanics, thoracolumbar fascia, tissue mechanics, musculoskeletal system

## Abstract

Spinal instability remains a complex phenomenon to study while the cause of low back pain continues to challenge researchers. The role of fascia in biomechanics adds to the complexity of spine biomechanics but offers a new window from which to investigate our spines. Specifically, the thoracolumbar fascia may have an important role in spine biomechanics, and thus the purpose of this study was to access the mechanical influence of the thoracolumbar fascia on spine biomechanics during different simulated activities. A numerical finite element model of the lumbar spine inclusive of the intra-abdominal and intra-muscular regions as well as the thoracolumbar fascia was constructed and validated. Four different loading scenarios were simulated while deformation, stress, pressure, and reaction forces between the thoracolumbar fascia and spine were measured. Model validation was accomplished through comparison to in vivo and ex vivo published studies. Force transmission between the thoracolumbar fascia and the spine increased 40% comparing kyphotic and squatting lifting patterns. Further, the importance of reciprocating paraspinal and intra-abdominal pressures was demonstrated. It was also found that tension in the thoracolumbar fascia remains even in a simulated prone position. This numerical analysis allowed for an objective interpretation of the loads conveyed through the thoracolumbar fascia in different positional or lifting scenarios. Based on validation studies, it would appear to be a viable experimental platform from which insight can be derived. The loads in the thoracolumbar fascia vary considerably based on simulated tasks and are linked to the pressures in the paraspinal and intra-abdominal regions.

## 1. Introduction

Spine stability, both approached from an engineering and clinical perspective, continues to elude researchers due to its high complexity and context-dependence. Mechanistically the spine is an indeterminate system controlled by different sub-systems, which forces researchers to make assumptions and narrow their scope of focus towards the study at hand. Nevertheless, important findings of spine functionality have consistently surfaced over the years. In addition to the well-accepted notion of muscle involvement in spine stability or control, intra-abdominal pressure (IAP) continues to show convincing importance. That is, an IAP increase may improve spine stability [1], while preloading or loading the spine may also increase its stability [2]. Even with musculoskeletal disorders, IAP and spine stability appear to be intertwined [3]. Moreover, while wearing lumbar belts to augment IAP without the need for further muscle contractions, one can improve spine stiffness [4]. Now how IAP converts to improved spine stability is believed to be associated with either anti-moment force vectors apposing flexion and/or the reciprocating and perhaps synergistic additional paraspinal contractions required to achieve such IAP [5,6,7]. The impact of IAP on the spine, regarding stabilization, at the cost or not of increased loading, appears to be activity-based or postural dependent [8,9]. Operating perhaps outside the conventional antagonist realm of offering stability or control is the notion that the muscular involvement in IAP creation is multifaceted, in the sense of also triggering alternative stabilization effects. An example of such is the role of the transverse abdominis in conveying forces, in the transverse plane, to the spinal process because of IAP, exemplified both via experimental [10] and in silico [11,12] platforms. 

The above summary only scratches the surface of the complexity of our spines. Moreover, the notion of stability reserves its interpretation, as in the present study, to engineering equilibrium amongst other equally important definitions. Often, you can gain knowledge of a system by studying its failure modes. The spine continues to be difficult, so to speak, as low back patients exhibit a myriad of symptoms while causative associations are near impossible. This is exasperated by the individuality of patients, each exhibiting different control strategies and the inherent mechanical properties of their stabilizing tissues. Hence, the more commonly used title of nonspecific low back pain, which better describes a symptom and not a condition. Even considering the heterogeneity element of nonspecific low back pain, sub-categorization proves slightly better in guiding treatment plans but does not present a path towards a panacea [13].

In silico or numerical platforms present an opportunity for researchers to explore biomechanical hypotheses, which are otherwise very complex or near impossible to execute via ex vivo or in vivo approaches. Regarding the spine, several notable groups leverage numerical analyses to conduct studies in spine biomechanics [14,15,16,17,18,19,20,21], while the research group of the present analysis seeks to focus their analyses on the involvement of fascia and adjacent pressures in spine biomechanics.

In accordance with the anatomical connectivity of the thoracolumbar fascia to the various spinal protrusions as well as past suggestions, such as Gracovetsky et al. [22] and Fairbank et al. [23], who suggested that forces may be transmitted between the transverse and axial directions to form of an extensor moment, a prior model [11,24] was further expanded to a 3D model in order to explore the out-of-plane effects of the Thoracolumbar Fascia in collaboration with the abdominal pressure. Specifically, this study seeks to investigate the axial force across the TLF using different boundary conditions or simulated patient positions.

## 2. Methods

**Figure 1:**
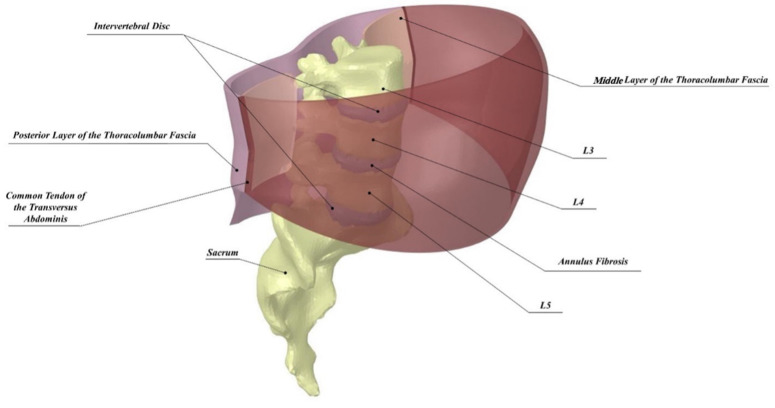
3D model of the lumbar spine with labeled anatomy.

The model geometry was constructed from a prior developed and validated 2D FE model [11], which was further developed from the same 3D MRI-based segmented patient data [25]. The present study included augmenting the planar section of this 2D model to a 3D model of the entire lower lumbar section (L_3_–S_1_). Further, the intervertebral discs were embedded between the vertebra and divided into annulus fibrosis and nucleus pulposus. Moreover, this 3D model included both the posterior and middle layers of the assumed two-layered TLF model connected to the abdominal wall layer via the common tendon of the Transversus Abdominis (cTrA). Figure 1 depicts the 3D model employed in this study. Data from 2 mm transverse planes were placed along the spine’s axial curvature for segmentation, and a 0.5 mm thickness was used to trace the planar linings of the abdomen and the TLF along with their respective compartments.

**Figure 2:**
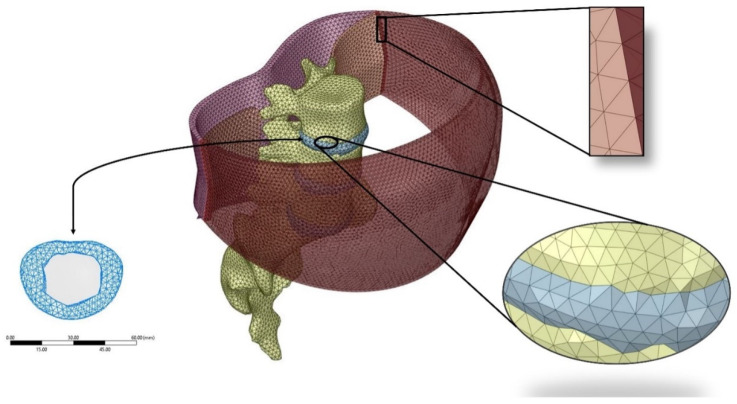
Demonstration of the meshed 3D spine model.

The numerical model and mesh were based on this full-scale three-dimensional geometric model, as described above. The planar linings were then blended together to achieve a 3D body. Next, the scoped components (shown in Figure 1) were all assembled in ANSYS Spaceclaim Design Modeler© (V18.1, Canonsburg, PA, USA), and the “Shrinkwrap” feature was utilized to generate a uniform mesh with an edge size of 0.5 mm. The open-source 3D graphics creation suite, Blender, was employed to merge the vertices together. The created surface mesh tessellation models were then re-imported into Spaceclaim with conforming vertices and edges. Then each of the surface meshes was converted to solid bodies without merging edges to complete the meshing workflow. The bodies were then imported into ANSYS Mechanical (V18.1, Canonsburg, PA, USA) for static structural analysis. Figure 2 shows a depiction of the resulting model and exemplifies the generated mesh and the resulting conformity between the different components. The resulting mesh was with a total of 188,961 tetrahedral elements and 272,442 nodes generated from the original unchanged tessellation surface mesh.

**Figure 3:**
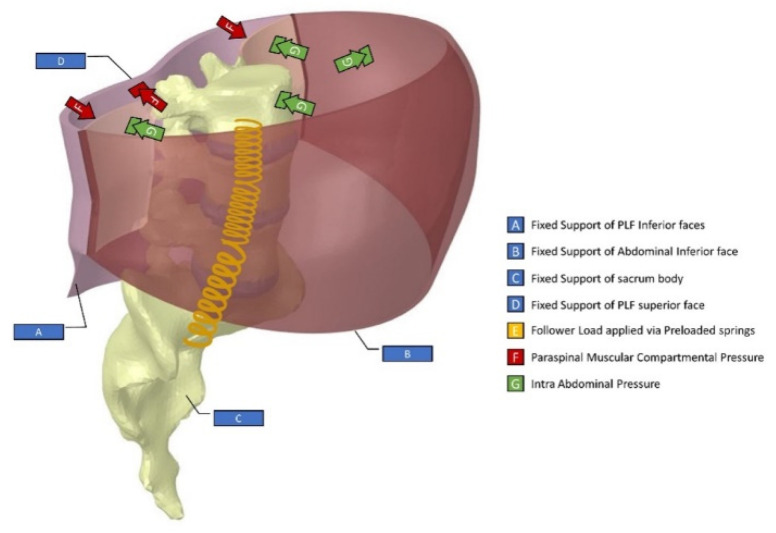
Boundary conditions of the model.

Material properties of the TLF (E = 450 MPa, ʋ = 0.4999), vertebra (E = 12000 MPa, ʋ = 0.3), and the abdominal wall (K = 2000 MPa, ʋ = 0.45) were employed. In addition, the cTrA was defined as linearly isotropic such that E = 1200 MPa and ʋ = 0.49 [26]. The intervertebral disc was defined as a linearly isotropic structure with an effective modulus of E = 5.7 MPa and ʋ = 0.37 for L_3_/L_4_ and L_5_/S_1_ [27]. However, the annulus fibrosis was defined as E = 4.2 MPa and ʋ = 0.45 and the Nucleus Pulposus was defined as E = 1 MPa and ʋ = 0.49 for the L_4_/L_5_ [28]. To account for degenerative disc progression in task 4, the nucleus pulposus was then changed to adopt mechanical properties as E = 1.35 MPa and ʋ = 0.32 while the annulus was maintained without change [27]. To account for the hydrostatic nature of the nucleus pulposus, HSFLD242, denoting 3D hydrostatic fluid elements, were adopted such that a pressure extended from the centroid of the object would be defined. This was conceived via an APDL command snippet added to the diagram tree, which further allowed the extraction of the intradiscal pressures (IDP). The boundary conditions designated in this study are shown in Figure 3. Four fixed supports were defined at the inferior and superior side of the Posterior Lumbar Fascia (PLF) layer, inferior side of the abdominal wall, and the sacrum. The follower loads (FL) were applied as 0 N for Task 1, 800 N (Upright posture) [29], 2350 N (Squatting posture carrying a 20 kg weight) [30], and 3185 N (Kyphotic Back Posture carrying a 20 kg weight) [30] for Task 2 and 144 N (prone posture) [29] for Tasks 3 and 4. The follower load was defined via 3 preloaded springs set at the centroids of the successive vertebra for all cases, as shown in Figure 3 and as modeled by others [31]. While the superior layer of the PLF deforms because of a force and/or moment effect, the side was fixed for probing the force/moment of the simulation. The intra-abdominal pressure and corresponding paraspinal muscle compartmental pressure inputs were varied correspondingly with details in results. 

A total of 32 named selections were defined within the model to identify the selected surfaces at which the loading was applied. Furthermore, large deflections were accounted for while force probes were set to capture the:

- Force Reaction at contact between PLF and spinous process of L_3_ (T_PLF/L3_);
- Force Reaction at contact between PLF and spinous process of L_4_ (T_PLF/L4_);
- Force Reaction at contact between PLF and spinous process of L_5_ (T_PLF/L5_);
- Force Reaction at fixed support of the superior side of the TLF (T_S_);
- Intradiscal Pressure of the L_4_/L_5_ Intervertebral Disc (IDP).

Simulations were selectively and sequentially performed, as detailed below, to allow for relative insight to be gained from the parameter controlled in silico environments building on prior validated models [11,12,24]. The following tasks were simulated:

1. A FE simulation was conducted by utilizing upright inputs, comprising 3.4 mmHg intra-abdominal pressure (IAP) and 18mmHg intra-muscular pressure (ICP) without any follower load, and compared the forces at the L3 level;
2. A FE simulation was performed utilizing (a) upright, (b) squatting with 20 kg weight, and (c) kyphotic back with 20 kg weight with a follower load of 800 N, 2350 N, and 3185 N. Extra validations of the disc stresses were performed.
3. A FE simulation was performed using the 3D model in a prone position with a follower load of 144 N. Extra validations of disc pressures were performed contrasting to healthy disc pressure of people without back pain.
4. A FE simulation utilizing the 3D model using prone position input specific to LBP cases with a Grade 3 degenerated disc with a follower load of 144 N and degenerated disc disease adjusted material properties.

## 3. Results

### Validation

The outputs showed that the 3D model PLF reaction forces were within close range of the 2D model [11], supporting the validation of the force output (Task 1). To ensure that the output results were applicable for the 3D model, the IDP was also compared with the literature. In specific, Task 2 showed that the upright posture resulted in an IDP value of 0.545 at the L_4_/L_5_ IVD, and Task 3 showed that the IDP in a prone position was 0.0986 MPa. In their study, Katsuhiko et al. investigated IDP in normal discs at the L4/L5 level in different postures in healthy individuals and reported an IDP of 0.539 ± 0.179 MPa in upright postures and 0.091 ± 0.027 and calculated the follower load to be 144 N and 800 N for the prone and upright postures, respectively [29]. Thus, the values achieved by Task 2 a) (upright) and Task 3 fall within the reported ranges when subjected to the same follower loads. Similarly, Wilke et al. reported on absolute values of IDP for different postures and exercises. In lifting a 20 kg weight with bent over round back (i.e., kyphotic back), the IDP was reported as 2.3 MPa, while lifting 20 kg as taught in back school (i.e., squatting), the IDP was reported 1.7 MPa with follower loads calculated as 3185 N and 2350 N, respectively [30]. In the case of this study, the resulting IDP for each of Task 2 b) (i.e., squatting) and Task 2 c) (i.e., Kyphotic Back) was 2.25 MPa and 1.606 MPa, corresponding to a 2.17% and 5.52% deviation, respectively. Hence, the IDP values recorded at L_4_/L_5_ fell within the reported values in the literature, serving towards model validation in this regard. 

**Figure 4:**
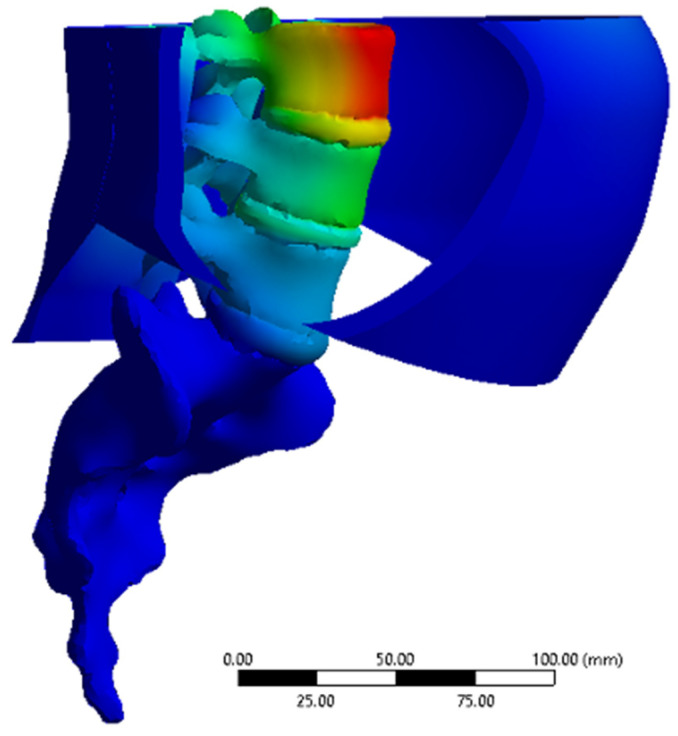
Deformation results example for task 3.

**Figure 5:**
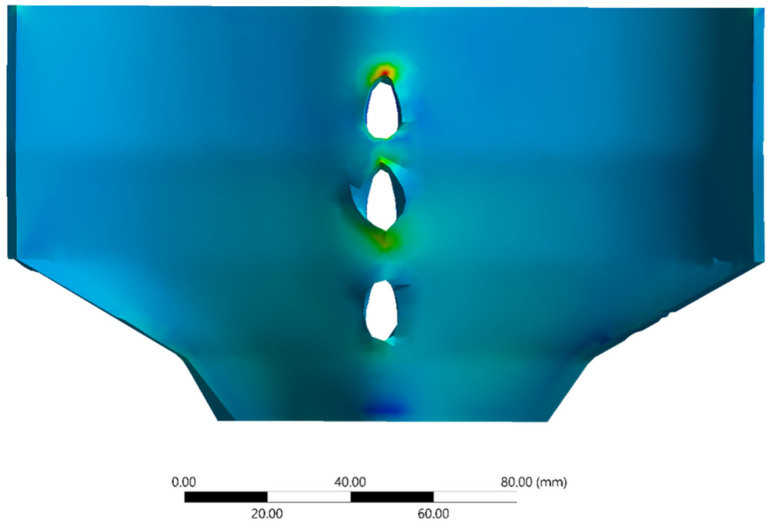
Shear stress distribution of the PLF layer for Task 3.

The average time per simulation was 31 seconds. Figure 4 depicts an example of the output deformation of the system (in mm) for Task 3 (normal prone posture). Figure 5 depicts the shear stress distribution of the PLF layer for Task 3 (normal prone posture). Table 1 reports the output values achieved for each of the four previously defined tasks.

Compared to upright position conditions, without a follower load (i.e., Task 1), the resulting posterior force at the L_3_ vertebral spinous process value increased by 363.71% when adding a follower load (800 N) in Task 2 (a) with an IDP of 0.545, which increased considerably in comparison with the IDP in Task 1. Moreover, the addition of a follower load increased the TLF axial reaction force by 784%. Squatting with a 20 kg weight achieved an output resultant of 31.5 N, 99.3 N, and 50.6 N for L_3_, L_4,_ and L_5_ PLF force at the spinous process, respectively, with an IDP value of 1.606 MPa. These values increase significantly by 37.31%, 35.13%, 40.28% for L_3_, L_4,_ and L_5_ PLF force spinal levels, respectively, when carrying a 20 kg load in kyphotic back posture. Moreover, the IDP at the L_4_/L_5_ IVD increased by 40.41%, and the TLF axial reaction force increased by 38.4%. In a prone position (i.e., Task 3), the posterior TLF forces decreased by approximately 80% for each spinal level and the TLF axial reaction force. In the LBP case (i.e., Task 4), the TLF forces decreased by 23% and 8.81% at the L_3_, L_4_ spinal level PLF reaction force, respectively, but increased by 3.18% for the L_5_ spinal level PLF reaction force. However, the axial reaction force increased significantly by 41.12%, and the IDP increased by 14.6%.

## 4. Discussion

This novel study, to the author’s knowledge, is the first to create and explore the influence of the TLF on 3D lumbar spine mechanics under different loading scenarios. The model showed validity and thus lends credibility to its use in exploring reaction forces between the TLF and spine. Moreover, results provided appropriately in vivo ranges of intervertebral disc pressures and trends in reported data agree with prior studies. Lastly, this study puts forth new insights towards spine biomechanics and the involvement of the TLF during different activities.

This study quantified the advantage of carrying weight under squatting postures in comparison with kyphotic postures. That is, results demonstrate the advantage in force transmission across all TLF peripherals within the L_3_–S_1_ when a squatting posture is adopted in lifting weight. This is especially evident in the high increase in axial TLF force (up to 350 N) when carrying a 20 kg weight in kyphotic back increases by 40% compared to that of a squatting posture accompanied with a reduction in IDP of approximately the same percentage.

This notion agrees with clinical studies that took in vivo measurement of disc pressures as well as the rule of thumb regarding clinical lifting recommendations suggesting squatting. Of further interest, the reaction loads between the TLF and spinous processes increased considerably between squatting and kyphotic lifting strategies solely on the premise that paraspinal and IAP pressures, as well as compressive loads, were altered to reflect inputs associated with this task. Thus, if a flexion or lumbar kyphosis was imposed, the displacement-imposed strain in the TLF would likely also lead to a significant increase in these reported reaction forces.

Second, this study quantified the axial TLF forces in the posterior layer under various postures in the lower lumbar region. Forces at the L5 level agreed with the prior literature [10]. Being a 3D study of multiple functional units in the lumbar spine, new appreciations of load sharing are reported. Although additional studies are required before being conclusive, results suggest that perhaps the TLF junction at L5 does not have the greatest TLF reaction loads in the transverse plane when compared to L4 and L3.

This study also reports on computed reaction forces, which arise from existing tensions in the TLF, provided by changes intra-muscular and intra-abdominal pressures (ICP and IAP). It was also shown that without reciprocating IAP pressure increase that the intra-muscular pressures on its own leads to the lateral rathe, junction between posterior and middle layers of the TLF, losing is tensional integrity and not providing a firm basis for reaction loads perhaps from movement in transverse plane [11]. This is perhaps due to the orientation of the reactive force vector confined to the tensional alignment of the tissue, which changes as a function of the lateral rathe position, which, in turn, is a function of the ICP and IAP ratios. This is also further observed in the present 3D study in which kyphotic lifting, having higher ICP and lower IAP compared to squatting, leads to a higher reaction loads at the spinous processes, perhaps resulting from larger paraspinal pressure and local muscle bulging forming without corresponding pull on the lateral rathe fromed from IAP. This notion will be further studied in subsequent studies. Interestingly, one may contrast another study in which not only positional loading, as observed herein, impacted fascia or TLF loading, and the geometry and mechanical properties impact what load it undertakes in the spine [24,32].

Lastly, this paper provides insight into resting tension in the TLF even when someone is prone and compares back vs. non-back pain. It is known spinal loading does not disappear when prone. 

To simulate this prone posture for a degenerated disc case, the material properties were adjusted accordingly in addition to the reported IAP and ICP inputs. In investigating the resultant outputs, the PLF reaction forces decreased more towards the higher spinal levels while the overall TLF axial pressure increased significantly (41.12%). This may indicate that the TLF tends to mismanage the force transmissions properly. In specific, the increase in IAP and ICP from an upright posture to higher demanding postures always resulted in an increase of all reaction forces. However, in the prone position case, the increase of IAP and ICP observed in LBP cases did not result in the same pattern (i.e., forces decreased for the L_3_ and L_4_ spinal levels as well as the fascia axial force but increased for the L_5_ spinal level). 

Like all numerical studies, one must be aware of limitations. It is worthy to note that the IDP increased by 14%; however, previous in-vivo studies observed a decrease in the IDP with the progression of the discal degeneration (0.073 ± 0.042 MPa in Grade 2 (mild) discal degeneration and 0.032 ± 0.045 in Grade 3 (moderate) discal degeneration [29]). This may be the result of not incorporating porosity to the nucleus pulposus characterization, whereby the decrease of IDP was suggested to be a result of the decrease in the water content. Moreover, the IDP discrepancy may be the result of the limitation of not accounting for the geometrical change in the nucleus pulposus, which was observed to have a decrease in height. Hence, further studies that incorporate the two prior variations are necessary. Furthermore, the model omits the inclusion of ligaments provided its movement is within the neutral zone in which ligamentous resistance can be considered negligible. Lastly, linear mechanical properties are used in this study in which physiological tissue is best defined as non-linear. This is justified by the model undergoing static analyses with a very little deformation in which may be considered to operate in the linear range of tissue, while non-linearities may most impact flexural and torsional loading.

Despite the above limitations, a careful effort was made to validate the model prior to interpreting data. Moreover, the insights put forth in the present study focus on relative results which compare numerical experiments in which variables can be fully controlled and hence subsequent results associated with confidence.

## Figures and Tables

**Table 1:** Model inputs and simulation results at different simulated positions.

			Task 1	Task 2			Task 3	Task 4
			FL = 0	FL = 800 N	FL = 2350 N	FL = 3185 N	FL = 144 N	
			Upright	(a) Upright	(b) Squatting with 20 kg	(c) Kyphotic Back with 20 kg	Prone (Normal)	Prone (LBP)
**Input**	**IAP**	mmHg	3.4	3.4	21.7	10.3	6.62	9.27
	**ICP**	mmHg	18	18	117	206	10	17.1
**Output**	**T_PLF_/L_3_**	N	2.72	12.613	31.526	43.291	2.293	1.77
	**T_PLF_/L_4_**	N	2.377	35.73	99.331	134.23	6.276	5.723
	**T_PLF_/L_5_**	N	1.786	17.031	50.67	71.081	3.61	3.725
	**Ts**	N	9.481	83.825	253.01	350.37	17.913	25.279
	**L_4_/L_5_ IDP**	MPa	0.00125	0.545	1.606	2.255	0.0986	0.113

## Data Availability

Not applicable.
